# Impact of an Intervention on Healthy Offerings and Allergenic Food Management in Restaurants: A Parallel Randomized Controlled Study

**DOI:** 10.3390/nu15234869

**Published:** 2023-11-22

**Authors:** Lucia Tarro, Floriana Mandracchia, Judit Queral, Maria Besora-Moreno, Nerea Vilanova, Rosa Maria Valls, Anna Pedret, Rosa Solà, Elisabet Llauradó

**Affiliations:** 1 Functional Nutrition, Oxidation, and Cardiovascular Diseases Group (NFOC-Salut), Facultat de Medicina i Ciències de la Salut, Universitat Rovira i Virgili, 43201 Reus, Spain; lucia.tarro@urv.cat (L.T.); floriana.mandracchia@outlook.it (F.M.); judit.queral@iispv.cat (J.Q.); mariadelaserra.besora@urv.cat (M.B.-M.); nerea.vilanova@urv.cat (N.V.); rosamaria.valls@urv.cat (R.M.V.); anna.pedret@urv.cat (A.P.); 2 Institut Investigació Sanitària Pere i Virgili (IISPV), 43204 Reus-Tarragona, Spain; 3 Hospital Universitari Sant Joan de Reus, 43204 Reus, Spain

**Keywords:** restaurants, healthy diet, food allergy, Mediterranean diet

## Abstract

The consumption of out-of-home meals is increasing. This study is aimed at assessing the effect of an intervention on healthy offerings and the management of food allergies and intolerances. Ten (control group) and eight restaurants (intervention group) were randomized in a 12-month parallel controlled trial. The outcomes were changes regarding adherence to the Mediterranean diet (AMed) and gluten management (SMAP) criteria, the traffic light rating category, nutrients, and gluten- and allergen-free content of dishes. After 12 months, and compared with baseline, there was an improvement of ≥25% in four items of the AMed criteria in the intervention group, whereas an increase in the offer of dairy desserts without added sugar, and a decrease in the first course offerings of vegetables and/or legumes were observed in the control group (*p* < 0.05). Also, after 12 months, there was an improvement of ≥50% in four SMAP criteria (*p* < 0.05) and in the mean average of all SMAP criteria (*p* = 0.021) compared with baseline in the intervention group, in which intra- and inter-group improvements for desserts in traffic light ratings, nutrients, and allergens were observed (*p* < 0.05). Therefore, the intervention showed beneficial effects, improving the quality of menus toward the Mediterranean diet pattern and gluten and food allergy/intolerance management.

## 1. Introduction

The consumption of out-of-home meals has increased in recent decades [1], with work and study commitments on weekdays and leisure and travel on weekends [2] being the main reasons. Out-of-home meals are linked to a negative impact on nutritional behavior [3,4]. On the one hand, eating out is associated with a higher consumption of energy, fats, and sodium and a lower consumption of fruits and vegetables [5]. These facts may increase exposure to overweight, obesity, and chronic diseases [6], raising morbidity and mortality risks [7]. On the other hand, eating away from home could be difficult for people with food allergies and intolerances. Food intolerance is a common feature affecting 15–20% of the population [8]. Around 2–37% of European adults have allergies to some food categories [9] and 1–19% to specific foods [10].

The implementation of healthier meal offerings in restaurants is a great opportunity to improve consumers’ dietary choices and promote healthy dietary habits. Additionally, in restaurants, changing to healthier offerings can increase customer satisfaction. In a cohort of restaurant consumers from ten countries worldwide, only 18% of them were satisfied with the healthy options available on menus [11]. Customers demand more vegetables, fresh ingredients, and light cooking from menus. In addition, customers are unsatisfied with the high price of food catered toward allergies/intolerances [11]. A total of 28% of fatal allergic reactions are linked to foods from restaurants or other establishments [12,13].

A systematic review of 25 studies on community-based restaurant interventions to promote healthy eating concluded that evidence is limited, and further studies with robust designs and standardized evaluation methods are needed [14]. Previous studies have shown that restaurants should improve the healthiness of their meals. Data from an observational study showed that US restaurant meals exceeded the American Heart Association’s (AHA) criteria concerning the median calories for total and saturated fats, cholesterol, and sodium. Overall, 22% of restaurant meals met zero to one of the AHA criteria, and only 8% met all seven of the AHA criteria [15]. A recent randomized cross-over trial with social norm messaging in retail store restaurants to reduce meat consumption demonstrated that the intervention had no effect [16].

In this scenario, a health intervention in restaurants is essential for improving both the food environment and individual eating behavior. A systematic review and meta-analysis on interventions promoting healthy meals in full-service restaurants and canteens was recently carried out [17]. The results showed the effectiveness of the interventions in school settings. However, the lack of randomized controlled studies in workplace and community settings, such as full-service restaurants, limited the evidence concerning the adult population and the evaluation of intervention effectiveness [17]. Due to this, the aim of the present study was to assess the effect of a multicomponent intervention in the context of a randomized controlled trial, applied to restaurant staff to promote a healthy diet and better management of food allergies and intolerances.

## 2. Materials and Methods

### 2.1. Study Design

A parallel, randomized controlled trial (The Healthy Meal Trial) was performed in restaurants in Tarragona (Spain). The trial was aimed at improving the nutritional quality of the menus offered and the availability of allergen-free dishes or those catering to specific food needs (i.e., vegan or vegetarian). The intervention period lasted 12 months. This study was part of a European-funded project called PECT-TurisTIC en Familia (The “Healthy Meals” operation) led by the Rovira and Virgili University (Tarragona, Spain). This study was conducted in accordance with the Declaration of Helsinki [18], and the protocol was approved by the Ethics Committee of the Institut d’Investigació Sanitaria Pere i Virgili (ref CEIM: 179/2018). The trial is registered in the international registry of clinical trials (ClinicalTrials.gov: https://clinicaltrials.gov/ct2/about-site/background, accessed on 21 February 2019) with the project identification code NCT03826576. All restaurant owners gave their informed consent for participation in this study. This study was conducted in accordance with the Consolidated Standards of Reporting Trials 2010 extension for randomized trials [19].

### 2.2. Study Population

**Figure 1:**
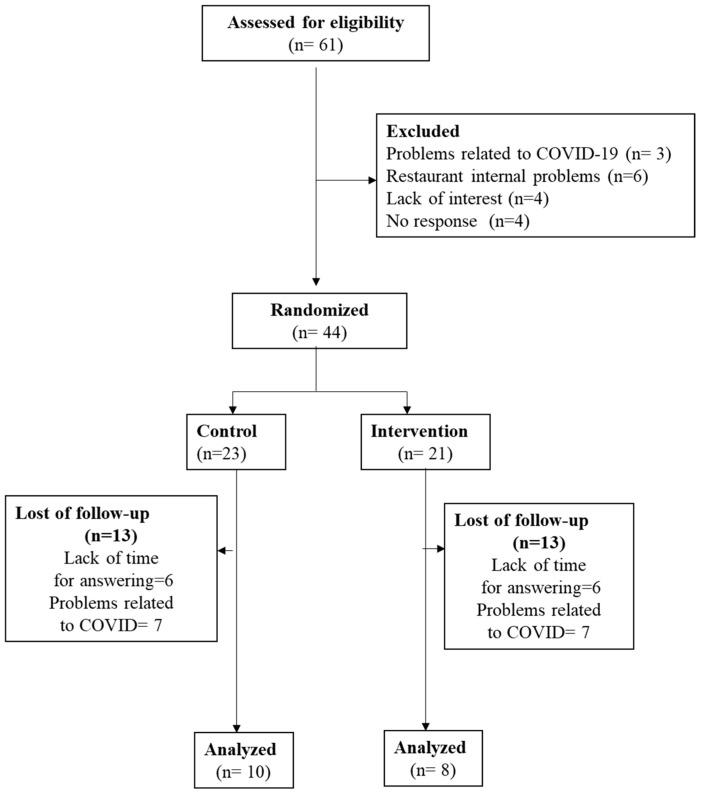
Flowchart of this study.

From September 2019 to March 2021, a total of 61 restaurants offering traditional and Mediterranean cuisine were recruited, and 44 of them were randomized as previously described [20] (Figure 1). Inclusion criteria were to (1) be a full-service restaurant; (2) have a minimum of 5 tables; (3) offer Mediterranean/traditional/local cuisine; (4) have technical details of the recipe for each dish, including ingredients and cooking details; (5) share food product information with the research team; (6) sign (the owner) an informed consent form for participation in this study; (7) have (the owner/s) a minimum of one year of experience; (8) plan to continue working during the full year of the intervention; and (9) not have a Mediterranean diet (AMed) certification. Exclusion criteria were ethnic or fast-food restaurants and failure to fulfill the above-described inclusion criteria.

### 2.3. Intervention

The multicomponent intervention included several actions:

- A virtual/face-to-face training for the kitchen and room staff on Mediterranean diet menu offers and food allergen management.
- A web-app to help chefs to improve their recipes concerning nutrition and allergen-free issues. Chefs can introduce each recipe of the menu in the app and be aware of the principal macronutrients (using a traffic light system) and the food allergens involved.
- Personalized recommendations for each restaurant based on key points for improving the Mediterranean diet profile and food allergen management.
- A marketing campaign about weak points to be improved in the restaurants concerning the two previously referred items.

### 2.4. Outcomes and Data Collection

The outcomes were directed at assessing changes from before to after the intervention.

#### 2.4.1. Adherence to the Mediterranean Diet Based on the AMed Criteria, Both Mandatory and Optional

AMed criteria are a list of items created by the Public Health Agency of Catalonia, Spain. AMed criteria are the basis for a certification to be provided to restaurants and food service establishments in order to guarantee the offer of a Mediterranean diet-based menu [21]. AMed items comprise nine mandatory and eight optional criteria. The mandatory criteria are (1) olive oil for dressings and olive oil or high-oleic sunflower for cooking; (2) 25% of the first-course offerings as vegetables and/or legumes; (3) presence of whole-grain products; (4) 50% of the second-course offerings based on fish, seafood, or lean meat; (5) 50% of the dessert offerings based on fresh fruit (whole or prepared); (6) offering dairy desserts without added sugar; (7) offering free nonpackaged drinking water; (8) wine, beer, and cava offered as glasses or individual units; and (9) culinary preparations that do not require the addition of large amounts of fat and culinary techniques with some or little fat.

#### 2.4.2. The Number of Gluten Management (SMAP) Criteria Fulfilled by Restaurants

The Malabsorption Syndrome Parents Association (SMAP) of the Catalan Celiac Association (Spain) has developed an SMAP certification [22]. SMAP consists of 18 recommendations for assessing gluten-free food management. The aim of the certification is to implement appropriate systems for cooking without gluten cross-contamination in restaurants to provide gluten-free dishes.

#### 2.4.3. The Number of Dishes Included in Each Traffic Light Category

From the nutritional information, a traffic light rating system for every dish was obtained, in agreement with the cut-offs provided by the UK Food Standards Agency [23]. The energy, carbohydrates, sugar, protein, total fat, saturated fat, and sodium, based on a single-plate portion, were classified as (a) green: the dish contains <7.5% of the nutrient amounts classified as unhealthy by the European Guideline Daily Amount (GDA) [24]; (b) orange: the dish contains between 7.5 and 20% of them; and (c) red: the dish contains >20% of them, for a healthy adult diet of 2000 Kcal. The fiber content was classified inversely so that a red label corresponded to a low fiber content according to the recommendations.

#### 2.4.4. The Nutrient Content of the Restaurant’s Dishes

The offered dishes were classified as starters, main dishes, and desserts. The information collected for each dish was energy (Kcal) and grams of protein, total carbohydrates, sugar, total and saturated fat, fiber, and sodium. The food composition database was extracted from the nutritional information of commercial food products and data from different public databases [25,26,27,28,29]. Nutritional information for each dish was based on the recipes including the cooking process, ingredients used, and their quantities.

#### 2.4.5. The Gluten and Allergen-Free Content of the Dishes Offered

The 14 most common food allergens that should be declared, according to the European Regulation 1169/2011 [23], were identified considering the ingredients used and the cooking process of the dishes: (1) cereals containing gluten, (2) milk, (3) eggs, (4) fish, (5) crustaceans, (6) tree nuts, (7) peanuts, (8) soya, (9) celery, (10) mustard, (11) sesame, (12) sulfites, (13) lupin, and (14) mollusks. The variable allergen-free was defined for dishes in which any of these allergens were present.

#### 2.4.6. The Offer of Vegetarian and Vegan Dishes

The typification of a vegetarian or vegan dish was made according to the ingredients used. The plant-based meals not containing animal products were labeled as vegetarian and the meals not containing animal products or their derivates were identified as vegan [30].

### 2.5. Data Analyses

Data are presented as mean ± standard deviation (SD) for continuous variables and as a percentage (number) for categorical variables. Differences in baseline characteristics between groups were assessed using Mann–Whitney or X^2^ tests. Wilcoxon and Mann–Whitney tests were used for intra- and inter-group comparisons, respectively, for categorical variables, and the X^2^ test was used for multiple comparisons. Student’s t-test was used for intra- and inter-group comparisons between related and unrelated samples, respectively, for continuous variables. Statistical significance was defined as a *p*-value ≤ 0.050 for a 2-sided test. Analyses were performed using SPSS for Windows, version 26 (IBM corp., Armonk, NY, USA). Before data collection, hypotheses and the analytic plan were specified, and any data-driven analyses were clearly identified and discussed appropriately.

## 3. Results

Of the 44 randomized restaurants, only 18 remained at the end of this study, 10 in the control group and 8 in the intervention group (Figure 1). Reasons for the loss of follow-up were related to the COVID-19 situation; specifically, seven control restaurants and six intervention restaurants closed due to the secondary effects of COVID-19, which included a lack of economic benefit. Another key reason was the availability of the restaurateur, who was too busy to spend time answering questionnaires.

### 3.1. General Characteristics

Table 1 shows the characteristics of the restaurants involved in this study. No differences between groups were observed in the type of restaurant, time of activity, frequency of change in the menu, and the type of cuisine or administration. The mean number of recruited employees, however, was higher in the control group when compared with the intervention group (*p* = 0.016).

### 3.2. Adherence to the AMed Criteria

Table 2 shows the adherence to the AMed criteria in control and intervention groups at the beginning and end of this study. Concerning mandatory criteria, in the control group, there was a decrease in the “25% of the first course offerings as vegetables and/or legumes” (from 50% to 10%) and an increase in “the offer of dairy desserts without added sugar” (from 20% to 60%) from the beginning to the end of this study (*p* < 0.05). In the intervention group, when considering both mandatory and optional criteria, increases of 37.5% were observed in “olive oil for dressing, and olive oil or high oleic sunflower for cooking” and “prioritize side dishes of vegetables and legumes”, but they did not reach significance (*p* = 0.083). In this group, a 25% increase was observed in the “offer of dairy desserts without added sugar” (*p* = 0.317) and “offer options with no added salt” (*p* = 0.157). No significant changes were observed in the other analyzed items or between treatments. When the average AMed criteria were compared, even though increases were observed after intervention in the AMed mandatory or in the sum of all AMed (compulsory and optional), neither intra nor inter-treatment significant differences were observed.

### 3.3. Gluten Management

Table 3 shows the gluten management in the control and intervention groups at the beginning and end of this study. In the control group, when comparing the beginning to the end of this study, the decrease from 70% to 20% in “preparation of gluten-free plates before the other food preparation” reached borderline significance (*p* = 0.057). In both groups, there was a 50% increase in “to place on the tables bottles of oil, vinegar, sauces, and baskets for exclusive use, or single-dose portions” (*p* = 0.046). In the intervention group, increases in “to provide a clean apron when working with flours or products that may leave gluten traces on the clothes” (from 37.5% to 70.5%, *p* =0.046) and in “do not use kitchen cloths and wooden tools, which are materials that can retain traces of gluten” (from 25% to 87.5%, *p* = 0.025) were observed. The increase observed in “to dispose of closed saltshakers and spice boxes or use a teaspoon to pick up the salt, as long as hands will be not placed inside” reached borderline significance (from 25% to 87.5%, *p* = 0.059). Additionally, in the intervention group, but not in the control group, the average of all SMAP criteria increased from the beginning to the end of this study (from 12.1 to 14.2, *p* = 0.021). No inter-treatment differences were observed.

### 3.4. Traffic Light Rating

**Figure 2:**
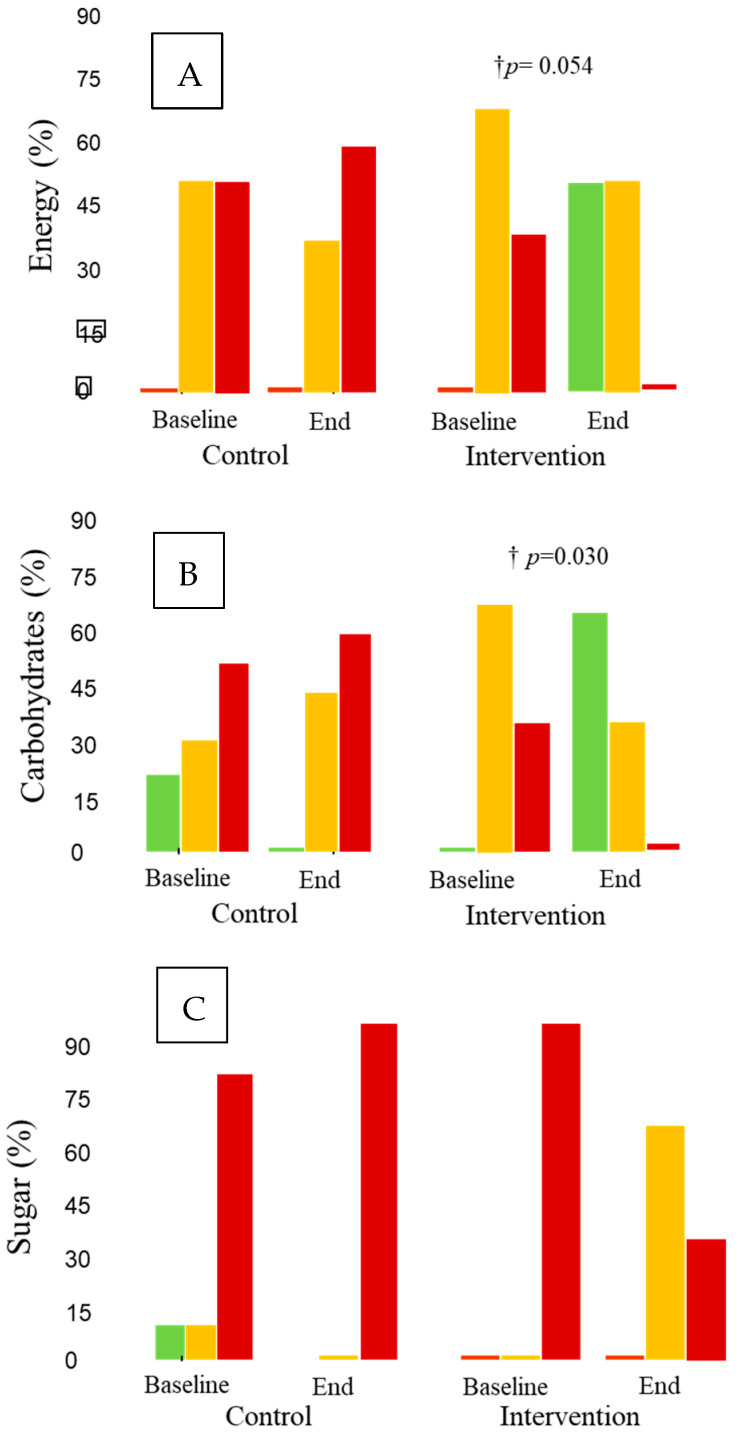
Traffic light ratings for desserts. † *p* for intra-treatment comparisons. Green: the dish contains <7.5% of the nutrient amounts classified as unhealthy by the European Guideline Daily Amount (GDA); orange: the dish contains between 7.5 and 20% of them; red: the dish contains >20% of them, for a healthy adult diet of 2000 Kcal. (**A**) Energy (%), (**B**) carbohydrates (%), and (**C**) sugar (%).

When the traffic light rating for restaurants was analyzed, neither intra- nor inter-treatment differences were observed for starters and main dishes (Supplementary Table S1). Concerning desserts (Figure 2), energy decreased, with a borderline significance (*p* = 0.054), from the beginning to the end of this study in the intervention group; the decrease reached significance compared with the changes observed in the control group (*p* = 0.017). A similar decreasing pattern in the intra-treatment changes for the intervention group was observed for carbohydrates (*p* = 0.030) and sugar (*p* = 0.016) (Figure 2). Neither intra- nor inter-treatment changes were observed for the other evaluated variables: total and saturated fats, proteins, sodium, or fiber.

### 3.5. Nutrient Content

**Figure 3:**
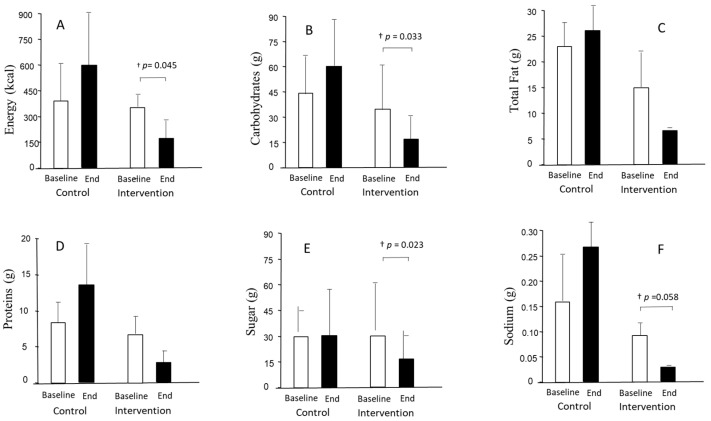
Nutrient content of desserts at the beginning and end of this study in the control and intervention groups. † *p* for intra-treatment comparisons. (**A**) Energy (kcal), (**B**) carbohydrates (grams), (**C**) total fat (grams), (**D**) proteins (grams), (**E**) sugar (grams), and (**F**) sodium (grams).

When assessing the nutrient content of the restaurants’ dishes, neither intra- nor inter-treatment differences were observed for starters and main dishes (Supplementary Table S2) with the exception of desserts. Figure 3 shows the nutrient content of desserts at the beginning and the end of this study in the control and intervention groups. A decrease was observed from the beginning to the end of this study in the intervention group for energy (*p* = 0.045), carbohydrates (*p* = 0.033), and sugar (*p* = 0.023), with the decrease in sodium values reaching borderline significance (*p* = 0.058). Despite considerable reductions in proteins or total fat after the intervention period, no significant differences were observed. No differences were observed in saturated fat or fiber values either. No inter-treatment differences were observed.

### 3.6. Gluten- and Allergen-Free Content of the Dishes and Vegetarian and Vegan Offers

**Figure 4:**
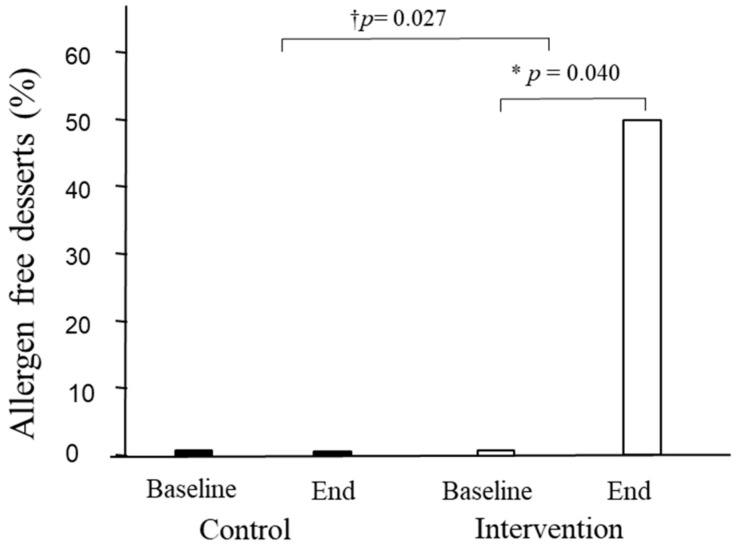
Percentage of desserts offered without allergens. * *p* for inter-treatment; † *p* for intra-treatment comparisons.

The gluten-free and allergen contents and vegetarian and vegan adequacy at restaurants were also examined. Neither intra- nor inter-treatment changes were observed in any variable for starters and main dishes (Supplementary Table S3). In desserts, however, the allergen-free content increased from the beginning to the end of this study in the intervention group (from 0% to 50%, *p* = 0.040), with the increase being significant compared with the changes in the control group (*p* = 0.027) (Figure 4).

## 4. Discussion

In this study, and after a 12-month intervention, improvements similar to or higher than 25% in four items of the AMed criteria in the intervention group were observed. In the control group, there was an increase from baseline data in the offerings of dairy desserts without added sugar (40%), with a decrease (40%) in the first course offerings as vegetables and/or legumes. Improvements of ≥40% in several SMAP criteria, as well as in the average of all SMAP criteria, were observed in the intervention group but not in the control group. Additionally, in the intervention group, but not in the control group, improvements in traffic light ratings, nutrients, and allergens were observed for desserts. Between-group differences were observed in energy reduction and allergen-free parameters in the intervention group desserts versus the control desserts. Thus, overall, the intervention showed beneficial effects that improved the quality of the menus.

In the intervention group, the main increases for AMed criteria were observed in relation to two items. The first item was “olive oil for dressing, and olive oil or high oleic sunflower for cooking”. A large body of knowledge exists on the advantages of olive oil consumption compared with other types of fats [31,32]. The improvement in this item has particular relevance and impact on the global nutritional quality of food offered, given that oils are involved in a large spectrum of common dishes such as salads or cooked vegetables, as well as in ways to prepare food (i.e., cooking meat and fish). The second item was “prioritize side dishes of vegetables and legumes”, in which an increasing trend was observed in the intervention group with a significant decrease in the control group. The prioritization of vegetable and legume side dishes implies a decrease in carbohydrate (i.e., white rice or pasta) consumption. The benefits of vegetable and legume consumption on health have also been largely documented [33,34].

Concerning SMAP criteria, the item “place on the tables bottles of oil, vinegar, sauces and baskets for exclusive use, or single-dose portions” significantly increased in both groups. This item was one of the mandatory conditions established for the partial reopening of restaurants in Spain in May 2020 after the COVID-19 pandemic lockdown [35]. Several SMAP criteria related to protection against gluten contamination (“to provide a clean apron when working with flours or products that may leave gluten traces on the clothes”; “do not use kitchen cloths and wooden tools, which are materials that can retain traces of gluten”; and “to dispose of closed saltshakers and spice boxes or use a teaspoon to pick up the salt, as long as hands will be not placed inside”) improved in both groups but reached significance only in the intervention group. Improvements in these items imply an active attitude toward avoiding gluten cross-contamination. Conversely, the “preparation of gluten-free plates before the other food preparation” decreased in both groups. This may be because it is time-consuming for the staff to accomplish this goal. It is important to remark that all SMAP criteria must have a gluten-free seal of quality, and their unfulfillment in restaurants could represent a huge problem for people with allergies when eating outside of the home. For this reason, as referred to in [36], it is important not only to use visible visual indicators but also to train staff about allergen awareness to avoid cross-contamination.

It is interesting that improvements in traffic light ratings, nutrients, and allergens occurred only in desserts in the intervention group, without changes in starters or main dishes. Despite having a small sample size, we were able to detect differences between groups for dessert energy regarding traffic light and allergen-free parameters. Regarding the traffic light ratings, there was an increase in green, a moderate decrease in orange, and a decrease in red for both energy and carbohydrates in the intervention group. Concerning sugar, however, the improvement in the intervention group was related to changes from red to orange, without modifications toward the green. Desserts are among the top five sources of fats and sugar [37]. Due to this, improvements in their nutritional value are noteworthy because there are few healthy dessert options [38], and desserts are usually consumed more outside of the home than at home [39]. In agreement with traffic light changes, changes in nutrients followed the same decreasing pattern for energy, carbohydrates, and sugar, but also for sodium. Improvements in meal energy represent one of the main goals for health. An observational study of 27 major UK chain restaurants on the energy content of their main meals showed it was excessive, not only in fast-food restaurants but also in sit-down restaurants [40]. Reducing calories in meals is a task for restaurants to accomplish, as giving consumers the option to choose, e.g., providing calorie information on the menu, seems to be relatively ineffective [41]. This also applies to reductions in sodium, given that consumers tend to underestimate its content in meals [42].

Strengths and Limitations

The present study has several strengths. First of all, this study’s design is robust enough to increase the available high-quality information on the effectiveness of this type of intervention. Additionally, focusing on the poor Mediterranean offerings on restaurant menus and difficulties preparing dishes without food allergens or with controlled food allergens is an original approach, considering the training of the restaurant staff and the provision of tools. This helps to increase their autonomy in improving the nutritional quality of the menu and food allergen detection, instead of teaching consumers about their selection when they visit restaurants.

One particular limitation of this study must be highlighted to explain the moderate success of the intervention and the timing of the intervention: from September 2019 to March 2021, the COVID-19 pandemic was particularly prevalent in Spain [43], with severe lockdown measures from March 2020 [44] to May 2020 [35]. During this time, restaurants could only trade through home delivery or takeaway. From May 2020 to February 2022, lockdown measures prompted restrictions both in the number of clients allowed in the establishment and in the opening hours [35,45]. In comparison with other industries, the restaurant industry has suffered the most significant sales and job losses since the COVID-19 outbreak began [46]. This also can explain the high loss of follow-up in this study, and the fact that the traffic light rating and nutrient improvements were only observed in desserts, as this was the easiest and cheapest way to make healthy changes to the menu. As a recently published article on the research impacts of COVID-19 points out, the COVID-19 pandemic placed a tremendous strain on sustaining the clinical research enterprise and will also likely affect key study outcomes; these effects must be considered during data analysis and interpretation [47]. The sample size reduction could have impaired the achievement of statistical significance between groups. Improvements were observed particularly in the intervention group despite a significantly low average number of recruited employees, with this fact reinforcing the benefits of the intervention. Another limitation was the difficulty in obtaining nutritional information about the dishes offered in restaurants. This is because, in the majority of cases, the restaurant staff did not have the recipes noted down in detail. The close contact that researchers had throughout this study with restaurateurs and staff personnel could explain the benefits obtained. Initiatives such as advertising campaigns, managed by town halls or local governments, directed at restaurants to improve their healthy offers are needed.

## 5. Conclusions

After a 12-month intervention, improvements were obtained in the intervention group for some AMed and SMAP criteria, as well as in traffic light ratings, nutrients, and allergens in desserts. The moderate rates of changes could be explained by the fact that this study was conducted in the context of the most severe COVID-19 pandemic period in Spain. Our results highlight the need for further interventions to enhance the nutritional quality and safety of menus offered in Spanish restaurants.

## Figures and Tables

**Table 1:** General characteristics of the restaurants.

	Control (*n* = 10)	Intervention (*n* = 8)	*p*
**Restaurant type, *%* (*n*)**			
Rural	20.0 (2)	25.0 (2)	0.610
Urban	60.0 (6)	37.5 (3)	
Coastal	20.0 (2)	37.5 (3)	
**Time of restaurant activity ^1^, *years***	9.5 (4.5–18.2)	4.0 (1.2–17)	0.360
**Frequency of menu change, % (*n*)**			
Twice a year (winter/summer)	20.0 (2)	25.0 (2)	0.804
More than twice a year	20.0 (2)	37.5 (3)	
The same through the year	40.0 (4)	25.0 (2)	
Other	20.0 (2)	12.5 (1)	
**Type of cuisine, % (*n*)**			
Traditional	50.0 (5)	62.5 (5)	0.596
Author	30.0 (3)	12.5 (1)	0.375
Fusion	20.0 (2)	0.0 (0)	0.180
Tapas	40.0 (4)	37.5 (3)	0.914
Other	0.0 (0)	12.5 (1)	0.250
**Administration of the restaurant, % (*n*)**			
The owner with her/his family	50.0 (5)	62.5 (5)	0.596
The owner with recruited staff	50.0 (5)	37.5 (3)	
Both previous items	0.0 (0)	0.0 (0)	
Recruited manager	0.0 (0)	0.0 (0)	
**Number of recruited employees ^1^**	7.0 (4.0–14.5)	4.0 (2.2–5.5)	0.016

^1^ median (25th–75th percentiles). *p* from Mann–Whitney or X^2^ tests.

**Table 2:** Adherence to the Mediterranean diet criteria (AMed1) in the control and intervention groups at the beginning and the end of this study.

	Control (*n* = 10)			Intervention (*n* = 8)			
AMed Criteria	Bas % (*n*)	End % (*n*)	*p* ^3^	Bas % (n)	End % (n)	*p* ^3^	*P* ^2,3^
1. Olive oil for dressing or cooking	90 (9)	90 (9)	1.0	62.5 (5)	100 (8)	0.083	0.237
2. First course is composed of 25% vegetable and/or legume offerings	50 (5)	10 (1)	0.046	50 (4)	62.5 (5)	0.317	0.101
3. Whole-grain products	20 (2)	10 (1)	0.564	37.5 (3)	37.5 (3)	1.0	0.762
4. Second course is composed of 50% fish, seafood, and lean meat offerings	50 (5)	60 (6)	0.564	62.5 (5)	62.5 (5)	1.0	0.762
5. Dessert is composed of 50% fresh fruit offerings	0 (0)	0 (0)	1.0	0 (0)	12.5 (1)	0.317	0.696
6. Dairy desserts without added sugar	20 (2)	60 (6)	0.046	50 (4)	75 (6)	0.317	0.762
7. Free nonpackaged drinking water	20 (2)	10 (1)	0.317	37.5 (3)	25 (2)	0.655	0.897
8. Wine or beer (offered as glasses)	100 (10)	100 (10)	1	100 (8)	100 (8)	1	1
9. Culinary preparations low in fat	100 (10)	100 (10)	1	100 (8)	100 (8)	1	1
**Total AMed mandatory criteria (mean ± SD) ^1^**	**4.50 ± 1.5**	**4.40 ± 1.3**	**0.798**	**5.0 ± 1.7**	**5.75 ± 1.0**	**0.320**	**0.276**
10. Fresh seasonal and local foods	100 (10)	100 (10)	1	100 (8)	100 (8)	1	1
11. Traditional and local cuisine	100 (10)	100 (10)	1	100 (8)	100 (8)	1	1
12. Virgin olive oil at the tables	90 (9)	80 (8)	0.564	100 (8)	87.5 (7)	0.317	0.965
13. Vegetables and legumes as side dishes	100 (10)	90 (9)	0.317	62.5 (5)	100 (8)	0.083	0.122
14. Point out to customers the most symbolic recipes	10 (1)	10 (1)	1	25 (2)	12.5 (1)	0.317	0.696
15. Unique dishes or medium portions options	80 (8)	70 (7)	0.317	62.5 (5)	62.5 (5)	1	0.762
16. No added salt options	90 (9)	100 (10)	0.317	75 (6)	100 (8)	0.157	0.633
17. Dissemination of nearby leisure activities	100 (10)	100 (10)	1	100 (8)	100 (8)	1	1
**Total AMed optional criteria (mean ± SD) ^1^**	**6.70 ± 0.82**	**6.50 ± 0.85**	**0.168**	**6.25 ± 0.71**	**6.62 ± 0.52**	**0.285**	**0.095**
**Total AMed criteria of 17 points (mean ± SD) ^1^**	**11.2 ± 1.93**	**10.9 ± 1.73**	**0.496**	**11.2 ± 1.91**	**12.4 ± 1.19**	**0.219**	**0.125**

Bas: Baseline. ^1^ AMed: Alimentacion Mediterranea (Mediterranean diet): criteria to obtain AMed certification (http://www.amed.cat/es/requisits.php, accessed on 17 November 2023), a ranking established by the Catalonian Government, Spain. ^2^ *p* for inter-treatment. ^3^ Wilcoxon tests and Mann–Whitney tests for intra- and inter-group comparisons, respectively. Student’s *t*-test for related and unrelated samples.

**Table 3:** Gluten management in the control and intervention groups at the beginning and the end of this study.

	Control (*n* = 10)			Intervention (*n* = 8)			
Criterium	Bas % (*n*)	End % (*n*)	*p* ^3^	Bas % (n)	End % (n)	*p* ^3^	*P* ^2,3^
1. Suppliers guarantee non-gluten	90 (9)	90 (9)	1.0	50 (4)	37.5 (3)	0.564	0.696
2. Store gluten-free products separately	100 (10)	90 (9)	0.317	62.5 (5)	75 (6)	0.564	0.515
3. Closed containers and content identified	80 (8)	100 (10)	0.157	100 (8)	100 (8)	1	0.515
4. Flours and breadcrumbs properly kept	70 (7)	100 (10)	0.083	75 (6)	87.5 (7)	0.317	0.573
5. Use different kitchen tools	100 (10)	100 (10)	1	100 (10)	87.5 (7)	0.317	0.696
6. Special equipment for gluten-free food preparation	40 (4)	40 (4)	1	37.5 (3)	50 (4)	0.564	0.696
7. Gluten-free plates prepared before other food	70 (7)	20 (2)	0.059	87.5 (7)	50 (4)	0.083	0.573
8. Differential area within the kitchen for gluten-free food	70 (7)	60 (6)	0.655	50 (4)	62.5 (5)	0.564	0.573
9. Cleaning before starting to work	100 (10)	100 (10)	1	100 (8)	100 (8)	1	1
10. Cleaning hands before preparation	100 (10)	100 (10)	1	100 (8)	100 (8)	1	1
11. Provision of a clean apron	60 (6)	90 (9)	0.083	37.5 (3)	87.5 (7)	0.046	0.515
12. Closed salt shakers and spice boxes, or a separate teaspoon to pick up the salt	40 (4)	70 (7)	0.083	25 (2)	87.5 (7)	0.059	0.203
13. Not using kitchen cloths and wooden tools	40 (4)	60 (6)	0.157	25 (2)	87.5 (7)	0.025	0.146
14. Not reusing oil, cooking water, or broths	100 (10)	100 (10)	1	75 (6)	100 (8)	0.157	0.408
15. Identification of dishes with gluten-free food	70 (7)	40 (4)	0.257	62.5 (5)	62.5 (5)	1	0.515
16. Re-preparing gluten-free dishes if a potential contamination occurs	100 (10)	100 (10)	1	100 (8)	100 (8)	1	1
17. Oil, vinegar, sauces, and baskets for exclusive use	10 (1)	50 (5)	0.046	25 (2)	75 (6)	0.046	0.762
18. Cleaning waiter’s hands before serving gluten-free dishes	100 (10)	100 (10)	1	100 (8)	100 (8)	1	1
**Average of all SMAP criteria (mean ± SD) ^1^**	**13.4 ± 1.78**	**14.0 ± 2.11**	**0.434**	**12.1 ± 2.23**	**14.2 ± 1.58**	**0.021**	**0.163**

Bas: Baseline. ^1^ According to the criteria of the Catalan Parents Association for the Malabsorptive Syndrome (SMAP, Sindrome Malabsortivo Asociación de Padres, (https://www.celiacscatalunya.org/ca/index.php), accessed on 17 November 2023). ^2^ *p* for inter-treatment. ^3^ Wilcoxon tests and Mann–Whitney tests for intra- and inter-group comparisons, respectively. Student’s *t*-test for related and unrelated samples.

## Data Availability

Emails can be directed to rosa.sola@urv.cat and elisabet.llaurado@urv.cat.

## Supplementary Materials

The following supporting information can be downloaded at: https://www.mdpi.com/article/10.3390/nu15234869/s1, Table S1: Traffic light ratings of the restaurants for starters and main dishes in the control and intervention groups at the beginning and the end of this study; Table S2: Nutrient content of the restaurants for starters and main dishes in the control and intervention groups at the beginning and the end of this study; Table S3: Allergen content, vegetarian, and vegan adequacy at restaurants throughout this study.

## References

[1] McGuffin L.E., Price R.K., McCaffrey T.A., Hall G., Lobo A., Wallace J.M.W., Livingstone M.B.E. (2015). Parent and child perspectives on family out-of-home eating: A qualitative analysis. Public Health Nutr..

[2] Diaz-Mendez C., Garcia-Espejo I. (2017). Eating out in Spain: Motivations, sociability and consumer contexts. Appetite.

[3] Lachat C., Nago E., Verstraeten R., Roberfroid D., Van Camp J.K.P. (2012). Eating out of home and its association with dietary intake: A systematic review of the evidence. Obes. Rev..

[4] Foundation B.N. British Nutrition Foundation. https://www.nutrition.org.uk/putting-it-into-practice/make-healthier-choices/healthy-eating-when-out-and-about/.

[5] Orfanos P., Naska A., Rodrigues S., Lopes C., Freisling H., Rohrmann S., Sieri S., Elmadfa I., Lachat C., Gedrich K. (2017). Eating at restaurants, at work or at home. Is there a difference: A study among adults of 11 European countries in the context of the HECTOR project. Eur. J. Clin. Nutr..

[6] Jayedi A., Soltani S., Abdolshahi A.S.-B.S. (2020). Healthy and unhealthy dietary patterns and the risk of chronic disease: An umbrella review of meta-analyses of prospective cohort studies. Br. J. Nutr..

[7] Organización Mundial de la Salud Enfermedades no Transmisibles Noncommunicable Diseases. https://www.who.int/news-room/fact-sheets/detail/noncommunicable-diseases.

[8] Kamran H., Imtiaz A., Amin F., Ghazzanfar S., Sani A., Fatima S., Aslam M., Jabeen S. (2020). Impact of food intolerance on quality of life among university students. J. Psychol. Clin. Psychiatry.

[9] Fernández-Rivas M., Barreales L., Mackie A.R., Fritsche P., Vázquez-Cortés S., Jedrzejczak-Czechowicz M., Kowalski M.L., Clausen M., Gislason D., Sinaniotis A. (2015). The EuroPrevall outpatient clinic study on food allergy: Background and methodology. Allergy.

[10] Lyons S.A., Burney P.G.J., Ballmer-Weber B.K., Fernandez-Rivas M., Barreales L., Clausen M., Dubakiene R., Fernandez-Perez C., Fritsche P., Jedrzejczak-Czechowicz M. (2019). Food Allergy in Adults: Substantial Variation in Prevalence and Causative Foods Across Europe. J. Allergy Clin. Immunol. Pract..

[11] Newson R., van der Maas R., Beijersbergen A., Carlson L., Rosenbloom C. (2015). International consumer insights into the desires and barriers of diners in choosing healthy restaurant meals. Food Qual. Prefer..

[12] Radke T.J., Brown L.G., Hoover E.R., Faw B.V., Reimann D., Wong M.R., Nicholas D., Barkley J., Ripley D. (2016). Food Allergy Knowledge and Attitudes of Restaurant Managers and Staff: An EHS-Net Study. J. Food Prot..

[13] Bailey S., Albardiaz R., Frew A., Smith H. (2011). Restaurant staff’s knowledge of anaphylaxis and dietary care of people with allergies. Clin. Exp. Allergy.

[14] Valdivia Espino J.N., Guerrero N., Rhoads N., Simon N.-J., Escaron A.L., Meinen A., Nieto F.J., Martinez-Donate A.P. (2015). Community-Based Restaurant Interventions to Promote Healthy Eating: A Systematic Review. Prev. Chronic Dis..

[15] Alexander E., Rutkow L., Gudzune K.A., Cohen J.E., McGinty E. (2020). Healthiness of US Chain Restaurant Meals in 2017. J. Acad. Nutr. Diet.

[16] Çoker E.N., Pechey R., Frie K., Jebb S.A., Stewart C., Higgs S.C.B. (2022). A dynamic social norm messaging intervention to reduce meat consumption: A randomized cross-over trial in retail store restaurants. Appetite.

[17] Mandracchia F., Tarro L., Llauradó E., Valls R.M. (2021). Interventions to Promote Healthy Meals in Full-Service Restaurants and Canteens: A Systematic Review and Meta-Analysis. Nutrients.

[18] World Medical Association WMA Declaration of Helsinki–Ethical Principles for Medical Research Involving Human Subjects. https://www.wma.net/policies-post/wma-declaration-of-helsinki-ethical-principles-for-medical-research-involving-human-subjects.

[19] Rennie D. (2001). CONSORT revised—Improving the reporting of randomized trials. JAMA.

[20] Mandracchia F., Llauradó E., Valls R.M., Tarro L., Solà R. (2021). Evaluating mediterranean diet-adherent, healthy and allergen-free meals offered in tarragona province restaurants (Catalonia, spain): A cross-sectional study. Nutrients.

[21] Agència de Salut Pública de Catalunya Generalitat de Catalunya. Amed Alimentació Mediterrània. http://www.amed.cat/requisits.php.

[22] Associació de Celíacs de Catalunya. https://www.celiacscatalunya.org/ca/establiment_acreditat.

[23] Food Standards Agency. Department of Health. Scotland Northern Ireland and Wales Governments Guide to Creating a Front of Pack (FoP) Nutrition Label for Pre-Packed Products Sold through Retail Outlets. https://www.gov.uk/government/publications/front-of-pack-nutrition-labelling-guidance.

[24] Rayner M., Scarborough P. (2004). The origin of Guideline Daily Amounts and the Food Standards Agency’s guidance on what counts as “a lot” and “a little. ” Public Health Nutr..

[25] RedBedca AESAN Base de Datos Española de Composición de Alimentos-BEDCA Spanish Database of Food CompositionBEDCA. https://www.bedca.net/.

[26] (2003). Mcgraw-Hill Tablas De Composicion De Alimentos Del Cesnid.

[27] Verdú J.M. (2003). Tabla De Composición De Alimentos. Food Composition Table.

[28] Favier J.C., Ireland-Ripert J., Toque C., Feinberg M. (1995). Répertoire général des aliments: Table de composition. General Food Directory: Composition Table.

[29] Moreiras O., Carbajal Á., Cabrera L., Cuadrado C. (2013). Tablas de composición de alimentos. Food Composition Tables.

[30] Tuso P.J., Ismail M.H., Ha B.P., Bartolotto C. (2013). Nutritional update for physicians: Plant-based diets. Perm. J..

[31] Gaforio J.J., Visioli F., Alarcón-de-la-Lastra C., Castañer O., Delgado-Rodríguez M., Fitó M., Hernández A.F., Huertas J.R., Martínez-González M.A., Menendez J.A. (2019). Virgin Olive Oil and Health: Summary of the III International Conference on Virgin Olive Oil and Health Consensus Report, JAEN (Spain) 2018. Nutrients.

[32] Pedret A., Fernández-Castillejo S., Valls R.M., Catalán Ú., Rubió L., Romeu M., Macià A., López de Las Hazas M.C., Farràs M., Giralt M. (2021). Cardiovascular Benefits of Phenol-Enriched Virgin Olive Oils: New Insights from the Virgin Olive Oil and HDL Functionality (VOHF) Study. Mol. Nutr. Food Res..

[33] Gibbs J., Cappuccio F. (2022). Plant-Based Dietary Patterns for Human and Planetary Health. Nutrients.

[34] English L.K., Ard J.D., Bailey R.L., Bates M., Bazzano L.A., Boushey C.J., Brown C., Butera G., Callahan E.H., de Jesus J. (2021). Evaluation of Dietary Patterns and All-Cause Mortality: A Systematic Review. JAMA Netw. Open..

[35] B.O.E (2020). Boletin Oficial del Estado (Spanish Official Journal). Saturday 9 of May of 2020. Disposition 4911. Article 16. https://www.boe.es/boe/dias/2020/05/09/pdfs/BOE-A-2020-4911.pdf.

[36] Barnett J., Begen F.M., Gowland M.H., Lucas J.S. (2018). Comparing the eating out experiences of consumers seeking to avoid different food allergens. BMC Public. Health.

[37] Wambogo E., O’Connor L., Shams-White M., Herrick K., Reedy J. (2022). Top sources and trends in consumption of total energy and energy from solid fats and added sugars among youth aged 2–18 years: United States 2009–2018. Am. J. Clin. Nutr..

[38] Craig W.J., Brothers C.J. (2022). Nutritional Content of Non-Dairy Frozen Desserts. Nutrients.

[39] Naska A., Katsoulis M., Orfanos P., Lachat C., Gedrich K., Rodrigues S.S., Freisling H., Kolsteren P., Engeset D., Lopes C. (2015). Eating out is different from eating at home among individuals who occasionally eat out. A cross-sectional study among middle-aged adults from eleven European countries. Br. J. Nutr..

[40] Robinson E., Jones A., Whitelock V., Mead B.R., Haynes A. (2018). (Over)eating out at major UK restaurant chains: Observational study of energy content of main meals. BMJ.

[41] Bailey R.L., Kwon K., Garcia C., Wang P. (2022). Fast food menu calorie labeling contexts as complex contributing factors to overeating. Appetite.

[42] Moran A.J., Ramirez M., Block J.P. (2017). Consumer underestimation of sodium in fast food restaurant meals: Results from a cross-sectional observational study. Appetite.

[43] Working group for the surveillance and control of COVID-19 in Spain, Members of the Working group for the surveillance and control of COVID-19 in Spain (2020). The first wave of the COVID-19 pandemic in Spain: Characterisation of cases and risk factors for severe outcomes, as at 27 April 2020. Euro Surveill.

[44] B.O.E Boletin Oficial del Estado (Spanish Official Journal). Number 67. https://www.boe.es/buscar/pdf/2020/BOE-A-2020-3692-consolidado.pdf.

[45] Instituto para la Calidad Turística Española (2020). Medidas para la Reducción del Contagio por el Coronavirus SARSCoV-2.

[46] National Restaurant Association USA. https://restaurant.org/education-and-resources/learning-center/business-operations/coronavirus-information-and-resources/.

[47] Tuttle K. (2020). Impact of the COVID-19 pandemic on clinical research. Nat. Rev. Nephrol..

