# Supplementary material for: Impact of an Intervention on Healthy Offerings and Allergenic Food Management in Restaurants: A Parallel Randomized Controlled Study
Source: Nutrients. 2023 Nov 22;15(23):4869. doi: 10.3390/nu15234869 (PMC10708017; doi:10.3390/nu15234869)
Supplement: Supplementary file 1 [file nutrients-15-04869-s001.zip › nutrients-2665362-supplementary.pdf]

Supplementary Table S1

Traffic light of the restaurants for starters and main dishes in control and intervention groups at the beginning and at the end of the study

	Control			Intervention			
	**Baseline %(n)**	**End % (n)**	**P**	**Baseline % (n)**	**End % (n)**	**P**	**P for inter-treatment**
**Starters**							
Energy			0.393			0.974	0.950
Green (2)	0 (0)	0 (0)		6.9 (2)	7.7 (2)		
Orange (1)	50 (5)	25 (1)		34.5 (10)	34.6 (9)		
Red (0)	50 (5)	75 (3)		58.6 (17)	57.7 (15)		
Protein							
Green	0 (0)	0 (0)	0.334	10.3 (3)	11.5 (3)	0.848	0.956
Orange	20 (2)	0 (0)		6.9 (2)	7.7 (2)		
Red	80 (8)	100 (4)		82.8 (24)	80.8 (21)		
Carbohydrates							
Green	60 (6)	25 (1)	0.442	44.8 (13)	53.8 (14)	0.865	0.946
Orange	20 (2)	50 (2)		41.4 (12)	34.6 (9)		
Red	20 (2)	25 (1)		13.8 (4)	11.5 (3)		

Supplementary Table S1 (cont)

Sugar							
Green	60 (6)	50 (2)	0.733	65.5 (19)	70.4 (19)	0.807	0.911
Orange	40 (4)	50 (2)		17.2 (5)	18.5 (5)		
Red	0 (0)	0 (0)		17.2 (5)	11.1 (3)		
Total Fat							
Green	0 (0)	0 (0)	0.217	3.4 (1)	3.7 (1)	0.898	0.690
Orange	30 (3)	0 (0)		27.6 (8)	22.2 (6)		
Red	70 (7)	100 (4)		69 (20)	74.1 (20)		
Saturated Fatty Acids							
Green	0 (0)	0 (0)		13.8 (4)	14.8 (4)	0.990	0.916
Orange	50 (5)	50 (2)	1.0	31 (9)	29.6 (8)		
Red	50 (5)	50 (2)		55.2 (16)	55.6 (15)		
Sodium							
Green	0 (0)	0 (0)	-	10.3 (3)	11.1 (3)	0.848	0.716
Orange	0 (0)	0 (0)		6.9 (2)	11.1 (3)		
Red	100 (0)	100 (4)		82.8 (24)	77.8 (21)		
Fiber							
Green	10 (1)	0 (0)	0.769	27.6 (8)	18.5 (5)	0.716	0.770

Supplementary Table S1 (cont)

Orange	60 (6)	75 (3)		34.5 (10)	40.7 (11)		
Red	30 (3)	25 (1)		37.9 (11)	40.7 (11)		
Main dishes							
Energy							
Green	6.3 (1)	11.1 (1)	0.894	4.9 (2)	6.5 (2)	0.954	0.897
Orange	31.3 (5)	33.3 (3)		22.0 (9)	22.6 (7)		
Red	62.5 (10)	55.6 (5)		73.2 (30)	71.0 (22)		
Protein							
Green	6.3 (1)	11.1 (1)	0.817	0 (0)	0 (0)	-	0.936
Orange	6.3 (1)	11.1 (1)		0 (0)	0 (0)		
Red	87.5 (14)	77.8 (7)		100 (41)	100 (31)		
Carbohydrates							
Green	62.5 (10)	77.8 (7)	0.432	36.6 (15)	32.3 (10)	0.890	0.991
Orange	37.5 (6)	22.2 (2)		36.6 (15)	41.9 (13)		
Red	0 (0)	0 (0)		26.8 (11)	25.8 (8)		
Sugar							
Green	81.3 (13)	77.8 (7)	0.835	85.4 (35)	90.3 (28)	0.545	0.713
Orange	18.8 (3)	22.2 (2)		9.8 (4)	3.2 (1)		

Supplementary Table S1 (cont)

Red	0 (0)	0 (0)		4.9 (2)	6.5 (2)		
Total Fat							
Green	0 (0)	0 (0)	0.918	0 (0)	0 (0)	0.658	0.648
Orange	12.5 (2)	11.1 (1)		24.4 (10)	29.0 (9)		
Red	87.5 (14)	88.9 (8)		75.6 (31)	71.0 (22)		
Saturated Fatty Acids							
Green	6.3 (1)	11.1 (1)	0.910	19.5 (8)	22.6 (7)	0.922	0.841
Orange	12.5 (2)	11.1 (1)		14.6 (6)	16.1 (5)		
Red	81.3 (13)	77.8 (7)		65.9 (27)	61.3 (19)		
Sodium							
Green	0 (0)	0 (0)	0.667	4.9 (2)	6.5 (2)	0.741	0.855
Orange	6.3 (1)	11.1 (1)		19.5 (8)	12.9 (4)		
Red	93.8 (15)	88.9 (8)		75.6 (31)	80.6 (25)		
Fiber							
Green	6.3 (1)	0 (0)	0.605	26.8 (11)	16.1 (5)	0.528	0.461
Orange	43.8 (7)	33.3 (3)		34.1 (14)	35.5 (11)		
Red	50.0 (8)	66.7 (6)		39.0 (16)	48.4 (15)		

P by X^2 test for intra- and inter-interventions

Supplementary Table S2

Nutrient content of the restaurants for starters and main dishes in control and intervention groups at the beginning and at the end of the study

	Control			Intervention		
	**Baseline**	**End**	**P**	**Baseline**	**End**	**P**
**Starters**	(n=10)	(n=4)		(n = 29)	(n = 27)	
Energy, *kcal*	402 (243-570)	521 (352-586)	0.394	424 (252-552)	420 (254- 543)	0.799
Proteins, *g*	18 (11-23)	19 (17-27)	0.570	18 (12-30)	17 (10-30)	0.583
Proteins, % of Energy	20 (12-26)	17 (13-23)	0.776	19 (12-30)	17 (9.7-29)	0.694
Carbohydrates,*g*	20 (10-48)	34 (20-68)	0.256	21 (8.3-41)	16 (7.4-31)	0.560
Carbohidrates, % of Energy	22 (11-36)	30 (19-48)	0.320	22 (8.4-32)	15 (7.2-25)	0.652
Sugar, *g*	6.5 (2.4-12)	7.3 (6.4-14)	0.394	4.5 (1.7-10)	4.5 (1.3- 8.2)	0.812
Sugar, % of Energy	4.6 (4.4-11)	7.5 (4.4-13)	0.719	4.1 (2.1-11)	3.9 (2.0-10)	0.831
Fat, g	19 (12-37)	26 (15-37)	0.570	25 (12-42)	25 (12-40)	0.993
Fat, % of Energy	55 (39-70)	50 (29-65)	0.670	60 (44-72)	68 (47-62)	0.527
Saturated Fat, *g*	3.6 (2.0- 11)	8.4 (2.4-19)	0.394	4.5 (1.9-14)	4.5 (1.9-14)	0.967
Saturated Fat, % of Energy	8.1 (6.3- 25)	17 (4.8-30)	0.776	8.9 (6.6-17)	9.0 (7.0-19)	0.743
Sodium, g	1.0 (0.8-1.5)	1.3 (0.8- 1.6)	0.478	1.0 (0.6-1.6)	0.8 (0.5- 1.4)	0.290

Supplementary Table S2 (cont)

Fiber*, g*	2.8 (1.6-3.9)	2.6 (0.5 -3.6)	0.776	2.4 (0.6- 5.1)	2.3 (0.4-3.4)	0.571
**Main dishes**	(n=16)	(n=9)		(n=41)	(n=31)	
Energy, *kcal*	480 (329-768)	527 (243-841)	0.978	505 (381-776)	450 (350-599)	0.397
Proteins, *g*	30 (25-44)	36 (14-56)	0.677	33 (23-44)	32 (22-39)	0.763
Proteins, % of Energy	24 (17-30)	26 (15-30)	0.978	25 (19-35)	28 (21-38)	0.328
Carbohydrates, *g*	11 (5.6-25)	10 (3.5-21)	0.357	21 (6.4-57)	21 (4.3-54)	0.789
Carbohydrates, % of Energy	10 (5.5-16)	8.1 (4.3-13)	0.357	12 (4.8-41)	13 (4.3-41)	0.973
Sugar, *g*	3.9 (1.4-5.6)	2.2 (1.1-5.5)	0.419	3.2 (1.2-5.6)	3.1 (1.2-5.2)	0.501
Sugar, % of Energy	2.7 (1.3-4.6)	1.6 (0.9-3.9)	0.335	2.6 (0.9-4.7)	2.8 (0.6-4.9)	0.815
Fat, g	25 (17-57)	32 (15-65)	0.934	25 (13-57)	23 (11-36)	0.423
Fat, % of Energy	59 (46-74)	60 (57-80)	0.357	50 (31-69)	48 (30-65)	0.484
Saturated Fat, *g*	6.8 (4.3- 16)	5.8 (3.8-18)	0.977	4.9 (2.4-11)	4.6 (1.7- 12)	0.621
Saturated Fat, % of Energy	13 (11-9)	16 (10-23)	0.541	10 (6.1-16)	11 (5.5-17)	0.955
Sodium, g	1.0 (0.8-1.8)	1.0 (0-7-1.2)	0.411	1.0 (0.5- 1.4)	1.0 (0.8-1.3)	0.968
Fiber*, g*	1.9 (1.4-3.9)	1.5 (0.2-2.1)	0.158	2.4 (0.7-4.4)	2.3 (0.4-2.9)	0.231

Data expressed as median (25th-75th percentiles). U Mann Whitney/W Wilcoxon test for intra-treatment comparisons. No between treatment differences were observed (Ps by X^2 =1)

Supplementary Table S3

Allergen content, vegetarian, and vegan adequacy at restaurants throughout the study

	**Control**			**Intervention**			
	**Baseline %(n)**	**End % (n)**	**P**	**Baseline % (n)**	**End % (n)**	**P**	**P for inter-Treatment**
**Starters**							
Gluten free	40 (4)	50 (2)	0.733	58.6 (17)	48.1 (13)	0.432	0.650
Vegetarian dishes	10 (1)	0.0 (0)	0.512	20.7 (6)	29.6 (8)	0.440	0.426
Vegan dishes	0.0 (0)	0.0 (0)	-	13.8 (4)	14.8 (4)	0.913	0.730
Allergen-Free	10 (1)	0.0 (0)	-	6.9 (2)	7.4 (2)	0.941	0.813
**Main dishes**							
Gluten free	62.5 (10)	44.4 (4)	0.383	51.2 (21)	45.2 (14)	0.611	0.363
Vegetarian dishes	0.0 (0)	0.0 (0)	-	0.0 (0)	0.0 (0)	-	-
Vegan dishes	0.0 (0)	0.0 (0)	-	0.0 (0)	0.0 (0)	-	-
Allergen-Free	6.3 (1)	11.1 (1)	0.667	2.4 (1)	3.2 (1)	0.841	0.716

Supplementary Table S3 (cont)

Desserts

Gluten free	70 (7)	71.4 (5)	0.949	50 (3)	37.5 (3)	0.640	0.605
Vegetarian dishes	60 (6)	42.9 (3)	0.486	83.3 (5)	75 (6)	0.707	0.611
Vegan dishes	0.0 (0)	0.0 (0)	-	16.7 (1)	50 (4)	0.198	0.122
Allergen-Free	0.0 (0)	0.0 (0)	-	0.0 (0)	50 (4)	0.040	0.027

P by X^2 test for intra- and inter-treatment changes
